# Association between morphologic grading and implantation rate of Euploid blastocyst

**DOI:** 10.1186/s13048-021-00770-8

**Published:** 2021-01-23

**Authors:** Hua Lou, Na Li, Yichun Guan, Yuchao Zhang, Dayong Hao, Shihong Cui

**Affiliations:** 1 Reproductive Center, the Third Affiliated Hospital of Zhengzhou University, Zhengzhou, 450052 Henan Province China; 2 Department of Obstetrics and Gynecology, the Third Affiliated Hospital of Zhengzhou University, Zhengzhou, 450052 Henan Province China

**Keywords:** Blastocyst morphologic grading, Day of trophectoderm biopsy, Euploid, Single frozen-thawed embryo transfer, Implantation rate

## Abstract

**Background:**

Standard morphologic evaluation has been the most widely adopted approach to embryo selection, and remains the most common strategy.The objective of the study to determine the association between the morphologic grading and implantation rate of euploid blastocysts in single frozen-thawed embryo transfer (SET) cycles.

**Methods:**

A total of 271 patients aged 20–40 years undergoing euploid SET from January 2017 to December 2019 were included in retrospective cohort study**.**The cycles were divided into three groups based on their morphologic grading before cryopreservation: good-quality (*n* = 58), average-quality (*n* = 88) and poor-quality blastocysts (*n* = 125). The pregnancy outcome of the three morphologic groups were analyzed and a logistic regression of implantation rate was conducted.

**Results:**

Good-quality blastocysts yielded statistically significantly higher implantation rates than poor-quality (79.31% vs. 48%; *P*<0.001). Planned subgroup analyses by age and the day of TE biopsy were conducted. Logistic regression analyses that adjusted for these variables identified higher implantation rates (adjusted odds ratio(aOR) = 4.083, 95% confidence interval (CI):1.836–9.082, *P*<0.001) for the good-quality blastocysts than for those that underwent poor-quality cycles in women aged < 35 years, but not in women aged ≥35 years (aOR = 6.074, 95% CI: 0.456–80.919, *P* = 0.172). The implantation rates were higher among women with good-quality blastocysts on both Day 5 and Day 6 of TE biopsy than among those with poor-quality blastocysts (Day 5, aOR = 3.294, 95% CI:1.260–8.616, *P* = 0.015; Day 6, aOR = 4.179, 95% CI:1.004 ~ 17.399, *P* = 0.049). Day 5 euploid blastocysts had no significant difference in implantation potential and early spontaneous abortion rate compared with similarly graded Day 6 euploid blastocysts.

**Conclusions:**

Blastocyst morphologic grading was associated with implantation rate for euploid embryo transfers after adjustment for potential confounders. These findings suggest that evaluating blastocyst morphology is critical when selecting the best euploid blastocyst.

## Background

The primary goal of assisted reproductive technology (ART) is to identify an embryo with a high implantation potential to achieve a single healthy live birth [1]. During the last few years, concerted efforts have focused on reducing multiple pregnancy after ART by restricting the number of embryos to transfer [2]. Therefore, one crucial step in an ART program is the selection of the embryo with the highest implantation potential [3]. Different strategies can be considered to meet this objective. One strategy involves culturing to blastocyst stage, thereby allowing the self-selection of embryos capable of proceeding to blastulation, together with microscopic evaluation of morphologic criteria associated with improved viability, such as trophectoderm, inner cell mass(ICM) and blastocoel expansion. Another strategy involves using preimplantation genetic testing for aneuploidy (PGT-A), mainly to reduce the likelihood of transferring an aneuploid embryo, which represents the leading cause of failed implantation, miscarriage and disordered embryo development [4].

PGT-A was originally applied as an embryo selection technique in women of advanced maternal age, those with recurrent pregnancy loss or patients with recurrent in vitro fertilization (IVF) failure [5]. Next-generation sequencing (NGS), which is the newest platform for PGT-A, performs high-throughput and high-resolution sequencing by synthesis. NGS is capable of generating large amounts of DNA sequence information both rapidly and at a low cost per base. Sequencing-based technologies are more cost effective and tend to have greater sensitivity than most other screening approaches [6].

However, even euploid embryos may fail to implant. The causes of failure warrant investigation. Embryo quality has always been considered an important predictor of successful implantation and pregnancy. Standard morphologic evaluation has been the most widely adopted approach to embryo selection, and remains the most common strategy [7]. Gardner and Schoolcraft’s morphologic parameter scheme entails expansion grades together with the individual evaluation of ICM and trophectoderm. A higher overall euploid blastocyst quality correlates strongly with optimal pregnancy outcomes. Thus, trophectoderm and ICM morphologic grades are likely to be effective supplementary parameters to consider during embryo selection [8]. Moreover, in a study by Irani et al. [9] evaluating the role of blastocyst development rate in euploid embryo selection, day 5 euploid blastocysts had higher implantation rates and live birth rates compared with similarly graded day 6 blastocysts.

Several aspects that could affect clinical embryo selection in euploid blastocysts should be considered. Therefore, we sought to determine whether the most important consideration is blastocyst morphologic grading or blastocyst development. The aim of this study was to assess the prognostic value of euploid blastocyst morphologic grading on the implantation competence.

## Methods

### Study design

The Third Affiliated Hospital of Zhengzhou University’s medical institutional review board approved this study. All patients attempting conception through intracytoplasmic sperm injection (ICSI) utilizing PGT-A at the Third Affiliated Hospital of Zhengzhou University Center for Reproductive Medicine from January 2017 to December 2019 were included. The inclusion criteria included a history of unsuccessful IVF attempts (≥3), and/or miscarriages (≥3), and/or advanced reproductive age (> 38 years), anyone of the three can be satisfied. Embryos that reached the blastocyst stage were biopsied and then vitrified to allow time for NGS analysis. All patients meeting the study criteria underwent SET of euploid blastocysts. The exclusion criteria were donor cycles, preimplantation genetic testing for monogenetic/single gene defects (PGT-M).

### Ovarian stimulation protocol

The GnRH antagonist protocol was preferentially used. After undergoing a baseline scan on the second or third day of the menstrual period, recombinant FSH (Gonal-F, Merck Serono, Switzerland) injections were started, the physician adjusted the starting dose (112.5 IU–300 IU) according to the patient’s age, body mass index (BMI) and ovarian reserve. Ovarian follicle development was monitored based on serum estradiol and transvaginal ultrasonographic measurements. The GnRH antagonist (Cetrotide, Merck Serono, Switzerland) at 0.25 mg/day was started on day 6 of stimulation or when the leading follicle reached 14 mm.Oocytes were retrieved transvaginal 33–36 h after the GnRH agonist 0.2 mg (Dophereline, Ipsen Pharma Biotech, France) was administered, which was done when at least 40% of follicles had reached or exceeded an average diameter of 18 mm as determined by ultrasound. The follicles were aspirated using a single-lumen needle attached to a syringe under transvaginal ultrasound guidance.

### Laboratory protocol

The oocytes were then inseminated via ICSI approximately 4 h after retrieval. Embryos were placed into the incubator (K-MINC-1000, Cook, United States) and cultured at 6% CO_2_, 5% O_2_ and 37 °C. G-1™ plus (Vitrolife, Sweden) was used to culture the embryos from the pronucleate stage to day 3, followed by G-2™ plus (Vitrolife, Sweden) from day 3 to the blastocyst stage.Embryologists graded the blastocysts on the degree of expansion and the morphology of ICM and TE according to the classification devised by Gardner,et al [10] The degree of expansion included the following six grades: 1: a non-expanded embryo with the blastocele filling < 50%; 2: the blastocele fills > 50% of the embryo; 3: the blastocele fills the entire blastocyst; 4: an expanded blastocyst with a thin zona pellucida; 5: a hatching blastocyst; and 6: a hatched blastocyst. The ICM was graded as follows: A: tightly packed cells; B: loosely gathered cells; and C: no identifiable cells. The ICM quality should be at least B in this study.The three TE grades were: A: many cells establishing a cohesive epithelial layer; B: few uneven cells creating a loose epithelium; and C: few large cells pushed to the side.According to the morphologic grading of euploid blastocysts: good-er-assisted trophectoderm (TE) biopsy was performed at the blastocyst stage on day 5 or 6. An opening of 6–9 μm was made in the zona pellucida with the use of a Saturn laser system (Research Instruments, Singapore), and 3–5 herniated TE cells were aspirated and separated from the blastocyst by applying multiple laser pulses. Harvested TE cells were loaded in 0.2-mL PCR tubes containing 5 μL phosphate-buffered saline solution (PBS) and stored at − 20 °C until further use. The biopsied embryos were screened for aneuploidy utilizing a targeted NGS-based PGT-A platform, as described in Zimmerman et al. [11] After biopsy, the blastocyst vitrificatioquality (AA, AB, BA), average-quality (BB) or poor-quality (AC, BC). Lasn was performed using Cryotop® (Kitazato Corporation, Shizuoka, Japan), as described previously [12].

### Next-generation sequencing

NGS technology (Thermo Fisher Scientific, United States) was used to analyze the TE biopsy samples. Whole genome amplification (WGA) was performed using SurePlex (Bluegnome, United Kingdom). Following WGA, the library preparation consisted of incorporating individual barcodes for the amplified DNA of each embryo. After isothermal amplification and enrichment, sequencing was performed in a 316 or a 318 chip using a Personal Genome Machine sequencer (Thermo Fisher Scientific, United States). Aneuploidy data analysis was performed using Ion Reporter software (Thermo Fisher Scientific), using the low-coverage whole-genome workflow. The blastocysts received a diagnosis of euploid, aneuploid or mosaic.

### Embryo transfer and clinical outcomes

Euploid SET was performed after preparation via hormone replacement treatment (HRT) or during a natural cycle. In the natural frozen embryo transfer (FET) cycle, women underwent monitoring by regular vaginal ultrasound combined with urine luteinizing hormone (LH) measurement to observe the development of the dominant follicle and endometrium from the 10th day of the menstrual cycle until ovulation. One euploid blastocyst was transferred 5 days after ovulation. In the HRT FET cycles, women received escalating doses of estradiol valerate (4 mg daily from the 3rd day of the menstrual cycle for 7 days, then 6 mg daily for 5 days). Endometrial thickness and pattern were monitored by vaginal ultrasound, and when the endometrial thickness was ≥7 mm, vaginal progesterone 900 mg/day (Crinone, Merck Serono, Switzerland) was provided for luteal support. On day 6 of progesterone administration, a single vitrified euploid blastocyst was selected for transfer based on morphologic grading. All of the transfer procedures were directed by ultrasound guidance as previously described [13].

The primary outcome measure for the study was implantation rate, defined as the proportion of transferred embryos whose implantation resulted in intrauterine gestational sacs visualized by transvaginal ultrasound. Early spontaneous abortion was considered to be a pregnancy that did not progress after a fetal heartbeat was seen on scan before 12 weeks of gestation.

### Statistical analysis

The baseline characteristics were compared with one-way analysis of variance for continuous data and Pearson’s chi-squared test or Fisher’s exact test for categorical data, as appropriate. A post hoc Bonferroni correction was applied for multiple comparisons. Multivariable logistic regression analysis was performed to detect the association between blastocyst quality and implantation rate. Odds ratios (OR) with 95% confidence intervals (CI) were calculated and adjusted for maternal age, BMI, type of infertility, basal FSH, AMH, peak endometrial thickness, type of SET cycle and day of TE biopsy. Planned subgroup analyses were conducted by age at oocyte retrieval (<35 years, ≥35 years) and the day of blastocyst biopsy (Day 5, Day 6). *P* < 0.05 was considered statistically significant. All of the data analyses were performed with SPSS software version 22.0 (IBM, United States).

## Results

### Baseline characteristics

A total of 271 frozen euploid blastocysts after transfer were included. The general outcomes of the cycles are reported in Table 1. The blastocysts obtained was 40.54% (750/1850), TE biopsy with NGS was performed in 660 blastocysts, 302(46.68%) of them were euploid, the other (53.32%) were aneuploid.

**Table 1:** Cycles utilizing preimplantation genetic testing for aneuploidy screening from January 2017 to December 2019 at a single infertility center

Characteristic	Description
Number of cycles	271
Mean female age(years, ±SD)	31.10 ± 3.74
Number of retrieved oocytes	3160
Mature oocytes (%)	2382/3160(75.38%)
Number of 2PN cleavage	1850/2382(77.67%)
Blastocysts obtained (%)	750/1850(40.54%)
Blastocysts biopsied (%)	660/750(88.00%)
Blastocysts with genetic results (%)	647/660(98.03%)
Aneuploid blastocysts (%)	345/647(53.32%)
Euploid blastocysts (%) Blastocysts no results(%) Clinical pregnancy (%) Ectopic pregnancy (%) Early spontaneous abortion (%)	302/647(46.68%) 13/660(1.97%) 163/271(60.15%) 3/163(1.84%) 23/163(14.11%)

The numbers of cycles classified into the three groups based on morphologic grading were as follows: good-quality blastocysts (*n* = 58), average-quality blastocysts (*n* = 88), poor-quality blastocysts (*n* = 125). The demographic parameters of patients in these groups are summarized in Table 2. There were no differences in age, BMI, type of infertility, basal FSH, AMH, peak endometrial thickness or type of SET cycle between the three groups. 79.3% (46/58) in the good quality group, 60.2%(53/88) in the average group and 41.6% (52/125) in the poor quality group had a transfer of Day 5 blastocysts, A post hoc showed that the distribution of embryo development stage at transfer differed significantly among embryo quality categories(both *P*<0.001).

**Table 2:** Demographic characteristics of patients who underwent frozen-thawed embryo transfer of euploid blastocysts according to morphologic grading

Parameter	Good-quality (*n* = 58)	Average-quality (*n* = 88)	Poor-quality (*n* = 125)	*P* value
Female age (y)	30.95 ± 3.50	31.64 ± 3.81	30.79 ± 3.78	0.253
Male age (y)	31.67 ± 3.76	32.49 ± 4.25	31.80 ± 4.54	0.417
Female BMI (kg/m^2^)	24.11 ± 3.42	23.96 ± 3.00	24.04 ± 2.79	0.952
Duration of infertility (y)	2.20 ± 1.95	2.68 ± 2.13	2.45 ± 1.98	0.364
Type of infertility				0.607
Primary infertility	17 (29.3%)	31 (35.2%)	46 (36.8%)	
Secondary infertility	41 (70.7%)	57 (64.8%)	79 (73.2%)	
Basal FSH (IU/L)	6.18 ± 2.55	6.58 ± 2.39	6.08 ± 2.32	0.314
AMH (ng/mL)	4.86 ± 4.01	4.60 ± 3.03	4.58 ± 3.09	0.854
Peak endometrial thickness (mm)	8.82 ± 1.52	9.05 ± 1.59	8.96 ± 1.56	0.687
Day of TE biopsy				0.000^*^
D5 (%)	79.3% (46/58)	60.2% (53/88)	41.6% (52/125)	
D6 (%)	20.7% (12/58)	39.8% (35/88)	58.4% (73/125)	

Note: Values are presented as mean ± standard deviation (SD) or number (%). *BMI* Body mass index, *AMH* Anti-Mullerian hormone, *FSH* Follicle-stimulating hormone, *TE* Trophectoderm

^*^P < 0.05 was considered statistically significant

### Primary outcomes

**Fig. 1:**
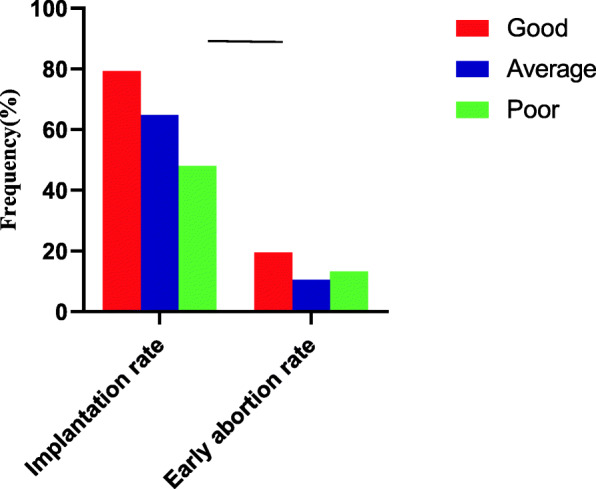
Implantation rates and early spontaneous abortion rates according to blastocyst morphologic grade.Good-quality blastocysts yielded statistically significantly higher implantation rates than poor-quality (*P* < 0.05)

Good-quality blastocysts yielded a statistically significantly higher implantation rate than poor-quality blastocysts (79.31% vs. 48.00%; *P <* 0.001; Fig. 1). There were no differences in implantation rate between the average- and poor-quality blastocysts (64.77% vs. 48.00%, *P* = 0.064). There were no differences in early spontaneous abortion rate among the three groups (*P* = 0.414).

### Secondary analyses

Planned subgroup analyses by age and the day of TE biopsy were conducted. The relationship between morphologic grading and implantation rates appeared to differ by a woman’s age. Logistic regression analyses that adjusted for these variables confirmed higher implantation rates (aOR = 4.083, 95% CI:1.836–9.082, *P* = 0.001) for the good-quality blastocysts than for those that underwent poor-quality cycles in women aged < 35 years, but not in women aged ≥35 years (aOR = 6.074, 95% CI: 0.456–80.919, *P* = 0.172) (Table 3).

**Table 3:** Implantation rates of euploid blastocysts categorized by female age and day of trophectoderm biopsy

Parameter	Category	No. of cycles	Implantation rate	aOR(95%CI)	*P* value
Age					
<35	Good	51	80.39% (41/51)	4.083(1.836 ~ 9.082)	0.001
	Average	69	63.77% (44/69)	1.768(0.941 ~ 3.324)	0.077
	Poor	109	48.62% (53/109)	Reference	Reference
≥35	Good	7	71.43% (5/7)	6.074(0.456 ~ 80.919)	0.172
	Average	19	68.42% (13/19)	4.304(0.608 ~ 30.472)	0.144
	Poor	16	43.75% (7/16)	Reference	Reference
Day of TE biopsy					
D5	Good	46	80.43% (37/46)	3.294(1.260 ~ 8.616)	0.015
	Average	53	71.70% (38/53)	2.062(0.871 ~ 4.855)	0.100
	Poor	52	55.77% (29/52)	Reference	Reference
D6	Good	12	75.00% (9/12)	4.179(1.004 ~ 17.399)	0.049
	Average	35	54.29% (19/35)	1.576(0.661 ~ 3.759)	0.305
	Poor	73	42.47% (31/73)	Reference	Reference

*Note:* Implantation rate adjusted for male/female age, BMI, type of infertility, basal FSH, AMH, peak endometrial thickness, type of SET cycle among the three groups. *BMI* Body mass index, *AMH* Anti-Mullerian hormone, *FSH* Follicle-stimulating hormone, *TE* Trophectoderm, *aOR* adjusted odds ratio, *CI* Confidence interval

*P* < 0.05 was considered statistically significant

Subsequent subgroup analysis revealed significant effect modification by the day of TE biopsy. On the same day as TE biopsy, morphologic grading was associated with the implantation rates of euploid blastocysts. Specifically, the implantation rates were higher among women with good-quality blastocysts on both Day 5 and Day 6 of TE biopsy than those with poor-quality blastocysts (Day 5, aOR = 3.294, 95% CI:1.260–8.616, *P* = 0.015; Day 6, aOR = 4.179, 95% CI: 1.004 ~ 17.399, *P* = 0.049).

**Fig. 2:**
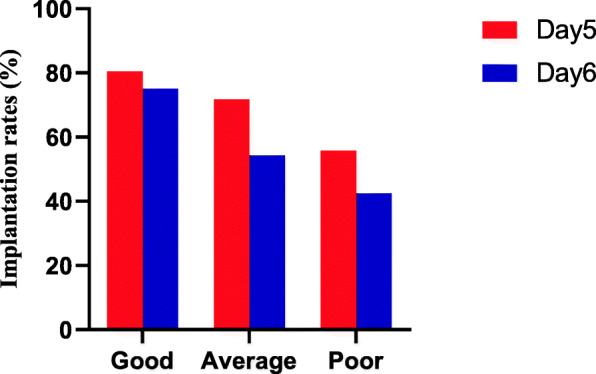
Implantation rates of different blastocyst morphologic grade that were biopsied on either day 5 or day 6. There was no significant difference in implantation rate among the 3 morphologic grade groups

**Fig. 3:**
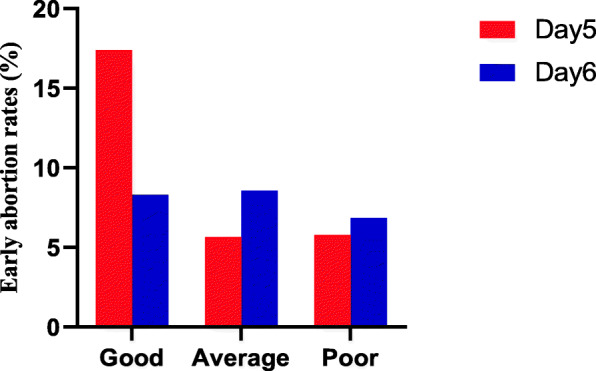
Early spontaneous abortion rates of different blastocyst morphologic grade that were biopsied on either day 5 or day 6. There was no significant difference in implantation rate among the 3 morphologic grade groups

Finally, to further assess the effects of blastocyst morphologic grading and the day of TE biopsy on implantation rate and early spontaneous abortion rate, we stratified the data by the day of TE biopsy, as shown in Fig. 2 and Fig. 3. In every morphologic grading group, there was no significant difference in implantation potential and early spontaneous abortion rate among similarly graded euploid blastocysts from Day 5 and Day 6.

## Discussion

To summarize, this study described the correlations between blastocyst morphologic grades and their implantation rates. Our findings indicated that good-quality euploid blastocysts had higher implantation rates than poor-quality blastocysts, although this increase was only apparent for patients under 35 years old. On the same day as TE biopsy, morphologic grading was associated with the implantation rates of euploid blastocysts. Day 5 euploid blastocysts showed no significant difference in implantation potential compared with similarly graded Day 6 euploid blastocysts. Unlike the implantation rates, however, the early spontaneous abortion rate was not correlated with blastocyst grading.

In this study, TE biopsy was performed at the blastocyst stage and NGS-based PGT-A was conducted to select euploid blastocysts for single vitrified-warmed blastocyst transfer. NGS has grown in popularity due to its ability to identify unbalanced translocations, segmental aneuploidies, some triploidies [6] and lower levels of mosaicism than other techniques [14]. Some research has found improved pregnancy outcomes and reduced spontaneous abortion rates with NGS due to the exclusion of these abnormal embryos [15]. However, the findings are not unanimous. In a multicenter randomized clinical trial [16] that performed PGT-A versus morphology as a selection criterion for single frozen-thawed embryo transfer in good-prognosis patients, PGT-A did not improve overall pregnancy outcomes for the cohort of women, as analyzed per embryo transfer or per intention to treat. It is possible that the detrimental effect of the biopsy pre-vitrification on the embryo viability may outweigh the benefit of PGT-A [17].

Despite the advances made with PGT-A, a considerable proportion of euploid embryos still fail to implant due to as yet unknown etiologies. In our study, the priority for euploid blastocyst selection for transfer was based on blastocyst morphology. Our data confirm that good-quality euploid blastocysts were correlated with higher implantation rates than poor-quality blastocysts, which partially explains some of the failed euploid cycles. Similarly, Irani et al. [18] has confirmed that blastocyst grading was statistically significantly correlated with the development potential of euploid embryos. Nonetheless, a total of 417 frozen embryo transfer cycles in which 477 embryos transferred were included. In our study, only single blastocyst transfer cycles were included to decrease the potential effects of blastocyst-blastocyst interactions.Two embryos within the uterine cavity may compete with each other for implantation and consequently result in a decreased implantation rate of each blastocyst.

The goal of using PGT-A is to avoid the useless transfer of aneuploid embryos and to limit he incidences of miscarriage and chromosomally abnormal pregnancies [19]. Minasi et al. [20] found that a greater likehihood of euploid among blastocysts with good morphology scores.Whether euploid blastocyst quality is related to spontaneous abortion rate.Here, we provide evidence that the early spontaneous abortion rate is not correlated with euploidy blastocyst grading. In contrast, a review of euploid blastocysts showed that blastocyst morphology, unlike the blastocyst development rate, affect spontaneous abortion rates suggest that abnormal embryo development, but not the pace of development, could be linked to higher spontaneous abortion rate [9].

Although aneuploidy is the most significant determinant of cycle outcome in the older population, an age-related decline in implantation occurs in euploid embryos, supporting the view that factors other than chromosome segregation errors play a role in age-related fertility decline [19].The mechanism for age-related decline in euploid implantation potential is unclear. Cimadomo et al. [21] showed an association with the prevalence of poor-quality blastocysts was the maternal age at oocyte retrieval. These results contradict previous findings in which the differences in outcomes based on age were nonsignificant after adjusting for embryo morphology [22]. After controlling for maternal age, here we provide evidence that good-quality euploid blastocysts have a higher chance of implanting than poor-quality blastocysts in women under 35 years; however, we found no differences in implantation rate based on blastocyst morphology in women over 35. The most likely explanation is that for women aged 35 or older, the most important feature associated with the euploid blastocyst implantation rate is the maternal age at oocyte retrieval rather than blastocyst morphology, which highlights the competence of poor-quality euploid embryos in women of advanced maternal age. This conjecture is supported by Gonzalez’s finding [23].

Our study demonstrated that good-quality euploid blastocysts yield higher implantation rates than same-day poor-quality blastocysts. Both Day 5 and Day 6 euploid blastocysts with good quality showed increased implantation ability, and no significant difference in implantation potential was found between similarly graded blastocysts from days 5 and 6. Shapiro et al. [24] found that Day 5 blastocysts were associated with higher clinical pregnancy rates than their Day 6 counterparts in fresh cycles, but had similar outcomes in FET cycles. While Day 5 and Day 6 embryos may have similar implantation potentials, the difference in the success rates observed in fresh cycles is essentially related to the suboptimal embryo-endometrial synchrony of Day 6 blastocysts. Contradicting our findings, Irani et al. [9] showed that Day 5 euploid blastocysts yielded higher implantation rates and live birth rates than similarly graded Day 6 blastocysts.In any event, morphologic grading is correlated with implantation potential, which is why it was chosen as the main criterion used to select the embryos for transfer in this study. In this regard, it is vital to provide patients with specific counseling focused on the evidence reported in the literature so they can make an informed choice.

This study has several strengths. First, it was specifically designed to find a strategy for selecting the best euploid embryo in patients who have multiple euploid embryos. Second, the chosen age range allowed the results to be clearly stratified by age. Third, we evaluated the role of the blastocyst morphology along with blastocyst development in euploid embryo selection. Fourth, as this study involved single euploid blastocyst transfer, all of the embryos were subjected to the same NGS platform protocols with uniform analysis of the PGT-A results. Our embryologists also used a consistent scoring system consisting of a number of standardized transfer parameters.

This study also has several limitations. First, being a retrospective study with a relatively small sample size and data collection from a single center, some bias was inevitable. Second, implantation rate, not live birth rate, was the primary outcome. Third, patients over 40 years of age were not included because of the risk of not having access to euploid blastocyst transplantation. This may affect the applicability of the clinical outcomes to older patients with different quality blastocysts. Fourth, although blastocyst morphology and blastocyst development were both explored, the study was not capable of identifying specifically which embryos were most able to be implanted successfully.

## Conclusions

This study confirms that embryo morphologic grading is correlated with implantation potential. If multiple embryos are euploid, morphology should be the main criterion used to select an embryo for transfer. However, the association between morphologic grading of blastocyst quality and implantation potential appeared to only hold in younger, not older women. On the same day as TE biopsy, morphologic grading was associated with the implantation rates of euploid blastocysts. Day 5 euploid blastocysts showed no significant difference in implantation potential compared with similarly graded Day 6 euploid blastocysts.

## Data Availability

The datasets used and/or analysed during the current study are available from the corresponding author on reasonable request.

## Abbreviations

SET: single frozen-thawed embryo transfer
ART: assisted reproductive technology
ICM: inner cell mass
PGT-A: preimplantation genetic testing for aneuploidy
IVF: in vitro fertilization
NGS: Next-generation sequencing
ICSI: intracytoplasmic sperm injection
PGT-M: preimplantation genetic testing for monogenetic
BMI: body mass index
PBS: phosphate-buffered saline solution
HRT: hormone replacement treatment
FET: frozen embryo transfer
LH: luteinizing hormone
OR: Odds ratios
CI: confidence intervals
